# Diagnostic Dilemma of Rounded Atelectasis in the Left Lower Lobe Showing High Uptake of 18F-Fluorodeoxyglucose: A Surgical Conundrum

**DOI:** 10.7759/cureus.83005

**Published:** 2025-04-25

**Authors:** Tomohito Saito, Yumiko Kono, Yuta Akahane, Natsumi Maru, Takahiro Utsumi, Aki K Kobayashi, Kento J Fukumoto, Hiroshi Matsui, Yohei Taniguchi, Haruaki Hino, Osamu Honda, Koji Tsuta, Tomohiro Murakawa

**Affiliations:** 1 Thoracic Surgery, Kansai Medical University, Hirakata, JPN; 2 Radiology, Kansai Medical University, Hirakata, JPN; 3 Pathology, Osaka Metropolitan University Hospital, Osaka, JPN; 4 Radiology, Himedic Clinic Nakanishima, Osaka, JPN; 5 Pathology, Kansai Medical University, Hirakata, JPN

**Keywords:** case report, comet tail sign, fluorodeoxyglucose, positron emission tomography, rounded atelectasis

## Abstract

Differentiating rounded atelectasis from lung cancer can be challenging. Rounded atelectasis has a low-to-moderate maximum standardized uptake value of^ 18^F-fluorodeoxyglucose (^18^F-FDG); however, some cases show high uptake, meaning that radiology-based diagnoses may not always be accurate. Herein, we report a rare surgical case of a patient with rounded atelectasis exhibiting considerable ^18^F-FDG uptake.

A 55-year-old man with a 37-pack-year smoking history was referred to our hospital for further investigation of an abnormal shadow in the left lower lung field. Chest computed tomography (CT) revealed a 45-mm solid tumor with bronchovascular convergence forming a “comet tail” sign in the left lower lung lobe. Positron emission tomography/CT with ^18^F-FDG showed increased uptake within a 30-mm region of the subpleural mass (SUVmax: 6.5). These findings necessitated a differential diagnosis to distinguish rounded atelectasis from lung cancer. The patient underwent video-assisted thoracoscopic left lower lung lobectomy with hilar lymph node dissection. Pathological investigation revealed granulomatous pleuritis and pneumonitis with no evidence of malignancy, consistent with rounded atelectasis. The patient had an uneventful postoperative course and was discharged six days after surgery. During a two-year follow-up period, no health-related issues, including lung cancer development, have been observed.

This rare case highlights the importance of a thorough investigation to exclude the possibility of lung cancer before confirming a diagnosis of rounded atelectasis in patients with pulmonary lesions exhibiting high ^18^F-FDG accumulation.

## Introduction

Rounded atelectasis is pathologically defined as a rounded region of collapsed lung tissue associated with an invaginated, fibrotic pleura and thickened, fibrotic interlobular septa [1]. Rounded atelectasis is more common in men (approximately 80% of cases), usually observed in the lower lung lobes, and characterized by converging bronchovascular markings (“comet tail” sign) on computed tomography (CT) [2]. Furthermore, ^18^F-fluorodeoxyglucose (^18^F-FDG) positron emission tomography (PET)/CT assists clinicians in differentiating rounded atelectasis from lung cancer because rounded atelectasis shows a low-to-moderate maximum standardized uptake value (SUVmax). However, recent reports have indicated that there are some metabolically active rounded atelectases with high ^18^F-FDG uptake, indicating that diagnoses based on radiological findings may not always be accurate [3,4]. Herein, we report a surgical case of rounded atelectasis with remarkable ^18^F-FDG uptake.

## Case presentation

**Figure 1:**
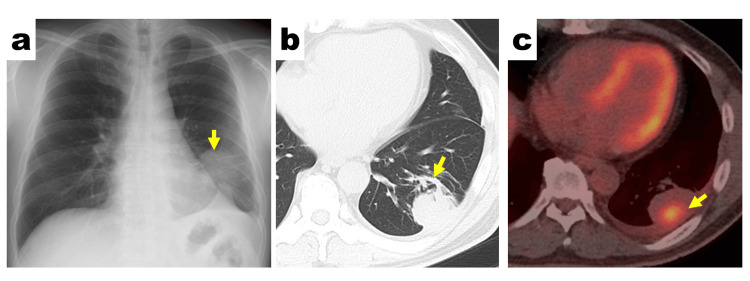
Preoperative radiological findings a. Chest radiograph showing an abnormal shadow in the left lower lung field (yellow arrow). b. Chest CT image revealing a 45-mm solid tumor with a comet tail sign (yellow arrow) in the left lower lung lobe (S9). c. Positron emission tomography with CT showing localized accumulation of ^18^F-FDG (yellow arrow). The maximum standardized uptake value of the lesion was 6.5. ^18^F-FDG: ^18^F-fluorodeoxyglucose; CT: computed tomography

A 55-year-old man with a 37-pack-year smoking history was referred to our hospital for further investigation of an abnormal shadow in the left lower lung field (Figure 1a). 

The patient had a history of gastroesophageal reflux disease. He was administered the coronavirus disease 2019 (COVID-19) vaccine Spikevax (Moderna Inc., Cambridge, MA, USA) four months before referral, after which he developed a high-grade fever associated with night sweats. Additionally, the patient reported a persistent cough for three weeks, which was triggered during meals. C-reactive protein levels and tumor markers, suc9h as carcinoembryonic antigen, cytokeratin fragment 21-1, squamous cell carcinoma, neuron-specific enolase, and progastrin-releasing peptide, were all within normal limits. Serological tests, including serum β-D-glucan, Candida mannan antigen, interferon γ-releasing assay for tuberculosis, and anti-Mycobacterium avium complex antibody were all negative (Table 1).

**Table 1:** Summary of preoperative laboratory test results of the presented case T-SPOT.TB: interferon γ-releasing assay for tuberculosis

Test	Patient value	Normal range
White blood cell count, ×10³/μL	6.6	3.0-8.5
C-reactive protein, mg/dL	0.047	≤0.30
Carcinoembryonic antigen, ng/mL	<1.0	≤5.0
Cytokeratin fragment 21-1, ng/mL	0.7	≤3.5
Squamous cell carcinoma antigen, ng/mL	0.5	≤1.5
Neuron-specific enolase, ng/mL	11.8	≤16.3
Progastrin-releasing peptide, pg/mL	50.6	<81.0
β-D-glucan, pg/mL	<6.0	≤20.0
Candida mannan antigen, U/mL	<0.02	<0.05
Cryptococcus antigen	Negative	Negative
Aspergillus antigen	Negative	Negative
T-SPOT.TB	Negative	Negative
Anti-Mycobacterium avium complex antibody, U/mL	<0.50	<0.70

Chest CT revealed a 45-mm subpleural mass showing a comet tail sign in the left lower lung lobe (S9) (Figure 1b). 18F-FDG-PET/CT showed increased 18F-FDG uptake within a 30-mm region of the subpleural mass (SUVmax, 6.5) (Figure 1c); no other abnormal uptake was observed. Brain contrast-enhanced magnetic resonance imaging showed no signs of brain metastasis. The lung tumor had CT findings characteristic of rounded atelectasis; however, the markedly increased 18F-FDG uptake suggested the possibility of lung cancer. Therefore, surgery was scheduled for suspected lung cancer (cT2bN0M0).

The patient underwent video-assisted thoracoscopic left lower lung lobectomy with hilar lymph node dissection under general anesthesia using the 37-French double-lumen endotracheal tube for one-lung ventilation. Paravertebral block patient-controlled analgesia was administered using the following regimen, as previously described: 500 μg of fentanyl in 10 mL, combined with 200 mL of 0.25% levobupivacaine and 90 mL of normal saline [5]. Intraoperatively, pleural adhesions were found, and the pulmonary tumor could not be excised by wedge resection owing to its anatomical location. Nodal dissection was limited to the hilar region because the gross appearance of the resected specimen suggested rounded atelectasis. The operation lasted 90 min, with intraoperative blood loss of 27 mL. During hospitalization, acetaminophen 750 mg was administered orally four times daily, and loxoprofen 60 mg was administered orally thrice daily.

**Figure 2:**
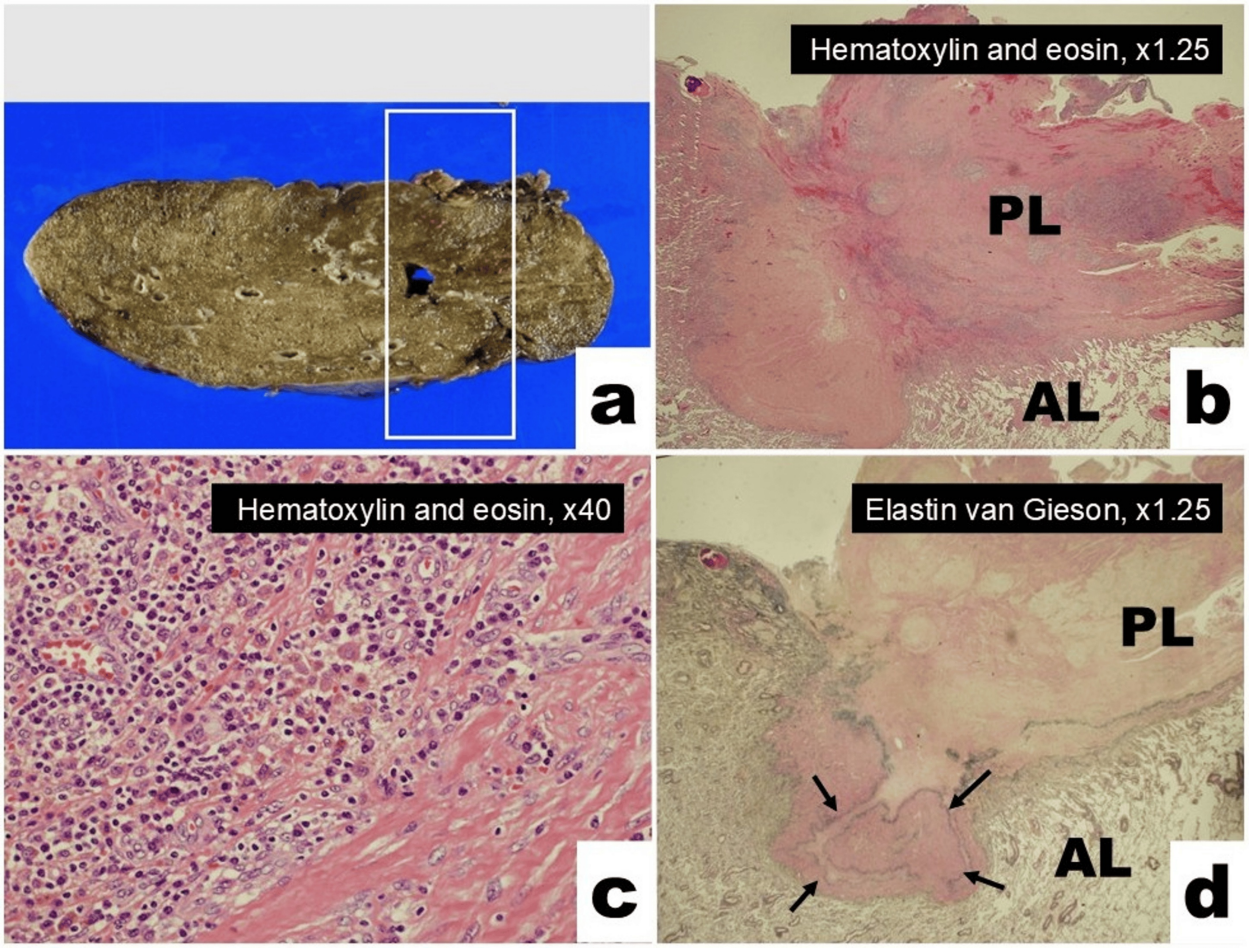
Pathological findings a. Macroscopic view of the pulmonary tumor showing no neoplastic appearance. b. Microscopically, the PL was thickened by fibrosis and granulomatous inflammation. Atelectatic fibrosis and calcification were evident within the AL area with no evidence of malignancy (Hematoxylin and eosin, ×1.25). c. Hemosiderin-laden macrophages were evident in the granulomatous inflammatory foci (Hematoxylin and eosin, ×40). d. The internal and external elastic lamina of the pleura showing invagination (black arrows) into the lung parenchyma, forming rounded atelectasis (Elastin van Gieson, ×1.25). PL: pleura; AL: alveolar

Macroscopically, the pulmonary tumor had no neoplastic appearance; however, a hard intrapleural nodule was palpable (Figure 2a). Microscopically, the pleura was thickened by fibrosis and granulomatous inflammation, including hemosiderin-laden macrophages (Figures 2b, 2c). The internal and external pleural elastic laminae were indented into the pulmonary parenchyma, forming the rounded atelectasis (Figure 2d). Atelectatic fibrosis and calcification were observed within the alveolar area, with no evidence of malignancy. Based on these findings, the pathological diagnosis was granulomatous pleuritis and pneumonitis, compatible with the clinical diagnosis of rounded atelectasis. Bacterial cultures were negative, and polymerase chain reaction tests for* Mycobacterium tuberculosis, Mycobacterium avium*, and *Mycobacterium intracellulare* were all negative. The patient was discharged uneventfully on postoperative day six. No signs of health-related problems including the development of lung cancer have been found during the two-year postoperative follow-up.

## Discussion

Two main etiological theories explain the formation of rounded atelectasis: the folding (pleural effusion) theory and the fibrosing (pleural injury) theory. The folding theory suggests that localized pleural effusion, commonly induced by pneumonia, compresses the lung, leading to subsequent invagination of the lung parenchyma [6]. The fibrosing theory suggests that local visceral pleural inflammation leads to fibrosis, resulting in the subsequent contraction of the fibrotic focus and the formation of rounded atelectasis [6]. In the present case, the patient's history and intrathoracic findings suggested preceding pneumonia, possibly owing to aspiration associated with gastroesophageal reflux disease; this is consistent with the “folding” theory. Based on the Vaccine Adverse Event Reporting System, pneumonia accounts for only 0.4% of all reported adverse events within two weeks of COVID-19 vaccination [7]. Therefore, the incidence of pneumonia and the subsequent development of rounded atelectasis associated with COVID-19 vaccination are estimated to be low.

Atelectasis can arise from both benign and malignant causes, with malignant origins being an important etiology [8,9]. Generally, rounded atelectasis can be clinically diagnosed based on CT findings; therefore, pathological confirmation is unnecessary as long as malignancy has been excluded. The comet tail sign on CT is considered pathognomonic for rounded atelectasis (sensitivity: 83% and specificity: 92%) [6]. However, as this sign can also be observed in lung cancer, follow-up imaging is necessary to differentiate rounded atelectasis from lung cancer [10]. Longitudinal CT studies have shown that 44% of rounded atelectasis lesions decrease in size over time, 44% remain unchanged, and 12% increase in size [11]. In the latter two scenarios, a differential diagnosis of lung cancer becomes crucial.

When CT findings are inconclusive, ^18^F-FDG-PET/CT is used to differentiate metabolically inactive rounded atelectasis from lung cancer due to its high sensitivity and moderate-to-high specificity [10,12]. The reported SUVmax of ^18^F-FDG-PET for atelectasis is 1.44±0.54, typically higher than that of normal lung parenchyma but generally lower than that of lung cancer [13]. Further, Cho et al. reported that benign obstructive lesions typically exhibit lower SUVmax compared to malignant obstructions (benign: 2.5±0.84; malignant: 11.8±5.95, p<0.001) [8].

An SUVmax of 4.0 yields a specificity of 96% in the diagnosis of rounded atelectasis [14]. Of note, however, the physiological ^18^F‑FDG uptake of normal structures in the thorax illustrates many benign pathological lesions with SUVmax >2.5 [15]. Thus, the results of ^18^F-FDG-PET/CT are meaningful only when the suspected lesion shows low ^18^F-FDG uptake because increased ^18^F-FDG accumulation can be associated with metabolically active rounded atelectasis and lung cancer. To date, five cases of rounded atelectasis with increased 18F-FDG uptake, including the present one, have been documented (Table 2) [3,4,16,17]. Notably, in two of these cases, the lesion size increased over time, and one case involved rounded atelectasis coexisting with lung cancer [16,17]. Therefore, patients with pulmonary lesions and features of rounded atelectasis showing ^18^F-FDG accumulation require thorough investigation to exclude the possibility of lung cancer.

**Table 2:** Summary of reported cases of rounded atelectasis showing increased 18F-FDG uptake CT: computed tomography; CTGB: computed tomography-guided biopsy; ^18^F-FDG: ^18^F-fluorodeoxyglucose; SUVmax: maximum standard uptake value; VATS: video-assisted thoracoscopic surgery

Authors, year	Age, sex	CT features of rounded atelectasis (e.g., comet tail sign)	Change in size over time	^18^F-FDG uptake	Pathological diagnosis
Norikane et al. 2020 [3]	64, M	Yes	N/A	SUVmax=9.6	Inflammatory change (CTGB)
Bae et al. 2015 [4]	53, F	Yes	N/A	SUVmax=2.7	Inflammatory change (CTGB)
Baral and Maskey 2018 [16]	77, M	Yes	Enlarged	SUVmax=4.0	Inflammatory change (CTGB)
Kadri et al. 2019 [17]	67, M	N/A	Enlarged	“Increased uptake”	Adenocarcinoma (CTGB)
Present case	55, M	Yes	N/A	SUVmax=6.5	Inflammatory change (VATS)

While CT-guided biopsy has high sensitivity (89-97%) and specificity (96-100%) for lung cancer, its negative predictive value (NPV) remains modest (51-88%) [18-20]. As CT-guided biopsy cannot completely rule out the possibility of lung cancer, surgical resection and subsequent pathological evaluation of surgical specimens should be considered when suspicion persists and the patient is deemed suitable for surgery. The video-assisted thoracoscopic approach may be an option, as randomized controlled trials have shown that video-assisted thoracoscopic lobectomy leads to better recovery of physical function at five weeks after randomization compared to open thoracotomy lobectomy [21].

The factors associated with metabolic activity in rounded atelectasis, as reflected by increased ^18^F-FDG uptake, remain unclear. Presumably, the granuloma within the rounded atelectasis lesion in the present case may be metabolically active, leading to false positivity for ^18^F-FDG-PET. Recent studies have revealed a metabolic transition toward glycolysis under hypoxic conditions and the utilization of the pentose phosphate pathway in granuloma-associated macrophages [22]. Consequently, the presence of granulomas may hinder the diagnostic accuracy of ^18^F-FDG-PET/CT in evaluating pulmonary nodules [23,24]. In hematology, ^18^F-fluorothymidine (FLT)-PET/CT shows greater capacity for differentiating malignancy from inflammation than ^18^F-FDG-PET/CT, with reduced false positives from inflammation [25]. This may be because ^18^F-FLT is an analog of thymidine, a nucleoside used in DNA synthesis, meaning that ^18^F-FLT accumulation correlates with cell proliferation. However, Norikane and colleagues reported that some rounded atelectasis shows ^18^F-FLT accumulation [3]. Additionally, fibroblast activation protein inhibitor (FAPI) is a novel imaging tracer targeting fibroblast activation protein in the tumor microenvironment [26]. Wei et al. showed that ^18^F-FAPI-PET achieves higher sensitivity (99% vs. 87%), specificity (93% vs. 79%), and NPV (97% vs. 70%) for lung cancer than ^18^F-FDG-PET [27]. Thus, FAPI-PET is a promising modality for further investigation in future studies. Nevertheless, we need to understand the limitations of the current ^18^F-FDG-PET/CT methods in differentiating malignancy from rounded atelectasis.

## Conclusions

This report details a rare surgical case of rounded atelectasis with granulomatous inflammation and increased ^18^F-FDG uptake. In cases of rounded atelectasis with ambiguous radiological features suspicious for lung cancer, such as increased ^18^F-FDG uptake, a pathological diagnosis based on surgical specimens is a reasonable option to differentiate rounded atelectasis from lung cancer.
